# Autophagy of macrophages is regulated by PI3k/Akt/mTOR signalling in the development of diabetic encephalopathy

**DOI:** 10.18632/aging.101586

**Published:** 2018-10-22

**Authors:** Beiyun Wang, Yuan Zhong, Qinjie Li, Liang Cui, Gaozhong Huang

**Affiliations:** 1 Department of Gerontology, Shanghai Jiao Tong University Affiliated Sixth People’s Hospital, Shanghai 200233, China; 2 Department of Priority Ward, Shanghai Jiao Tong University Affiliated Sixth People’s Hospital, Shanghai 200233, China; * Equal contribution

**Keywords:** diabetes, diabetic encephalopathy (DE), inflammatory macrophages, autophagy, mTOR

## Abstract

The development of diabetic encephalopathy (DE) is enhanced by inflammatory macrophages, and is suppressed by macrophage autophagy. However, the molecular signaling that controls macrophage autophagy in DE remains ill-defined. Here, DE is induced in rats that received intraperitoneal injection of streptozotocin (STZ). In macrophages isolated from the brain of the rats, we detected downregulated autophagy activity and enhanced PI3k/Akt/mTOR/S6K1 signaling. In order to examine the role of autophagy and PI3k/Akt/mTOR signaling in DE development, an mTOR inhibitor, rapamycin, or an autophagy inhibitor, chloroquine (CQ), were administered to the rats that that received STZ. We found that rapamycin significantly enhanced DE development through mTOR suppression-induced augmentation of macrophage autophagy, while CQ significantly decreased DE development through suppression of macrophage autophagy. Together, our data suggest that PI3k/Akt/mTOR signaling may promote the development of DE through suppression of macrophage autophagy.

## Introduction

Impairment of central nervous system function often occurs as a severe complication of diabetes [1]. Past studies have shown that diabetic encephalopathy (DE) is caused primarily by constitutive hyperglycemia [2], although recent evidence suggests that oxidative stress and brain inflammation promote the development of DE and associated neurodegenerative diseases [3–7], highlighting a specific role for activated infiltrating macrophages in disease progression [8]. Recently, we reported that DE could be suppressed through depletion of inflammatory macrophages, without affecting the underlying diabetic status [9]. However, the molecular regulation of inflammatory macrophages in diabetes is unknown.

Autophagy comprises the phagocytic degradation of lysosomes, not only as a recycling system within the cell, but also as an important defense mechanism of the body [10]. Conjugation of cytosolic microtubule-associated protein 1A/1B-light chain 3 (LC3-I) to phosphatidylethanolamine LC3-phosphatidylethanolamine conjugate (LC3-II) accompanies autophagosome formation and activity. The ratio of LC3-II to LC3-I levels reliably represents the level of autophagy activity [11–15]. In macrophages, autophagy status influences the cell’s specific function. Macrophages bind to lysosomes through autophagy, eliminating, degrading and digesting damaged, degenerated, senescent and dysfunctional cellular components: cells and organelles, denatured proteins, nucleic acids and other biological macromolecules [16]. This function provides essential raw material for cellular reconstruction, regeneration and repair, enabling the recycling of intracellular resources [16]. mTOR signaling is not only an important factor regulating cell growth and proliferation, but is also a key regulator of the autophagy initiation phase and can inhibit autophagy after activation [17–19]. The type I PI3K and Akt/PKB pathways are delivered to mTOR complex 1 (mTORC1) which, in turn, drives the activation of mTORC1, and subsequently activates mTOR to inhibit autophagy [20]. The classical mTOR inhibitor is rapamycin (RAP) [21], which also acts as an activator of autophagy. Chloroquine (CQ) prevents autophagosomal degradation in late phase autophagy [22].

In this study, we aimed to investigate the molecular signaling that controls macrophage autophagy in DE. DE is induced in rats that received i.p. injection of streptozotocin (STZ). In inflammatory macrophages isolated from the brain of the rats, we detected downregulated autophagy activity and enhanced PI3k/Akt/mTOR/S6K1 levels. In order to examine the role of autophagy and PI3k/Akt/mTOR signaling in the development of DE, an mTOR inhibitor, RAP, or an autophagy inhibitor, CQ, were administered to the rats that that received i.p. injection of STZ. The rats were then assessed for DE development and levels of macrophage autophagy. Our results suggest that PI3k/Akt/mTOR signaling promotes the development of DE through suppression of macrophage autophagy.

## RESULTS

### Development of DE in rats after STZ

**Figure 1:**
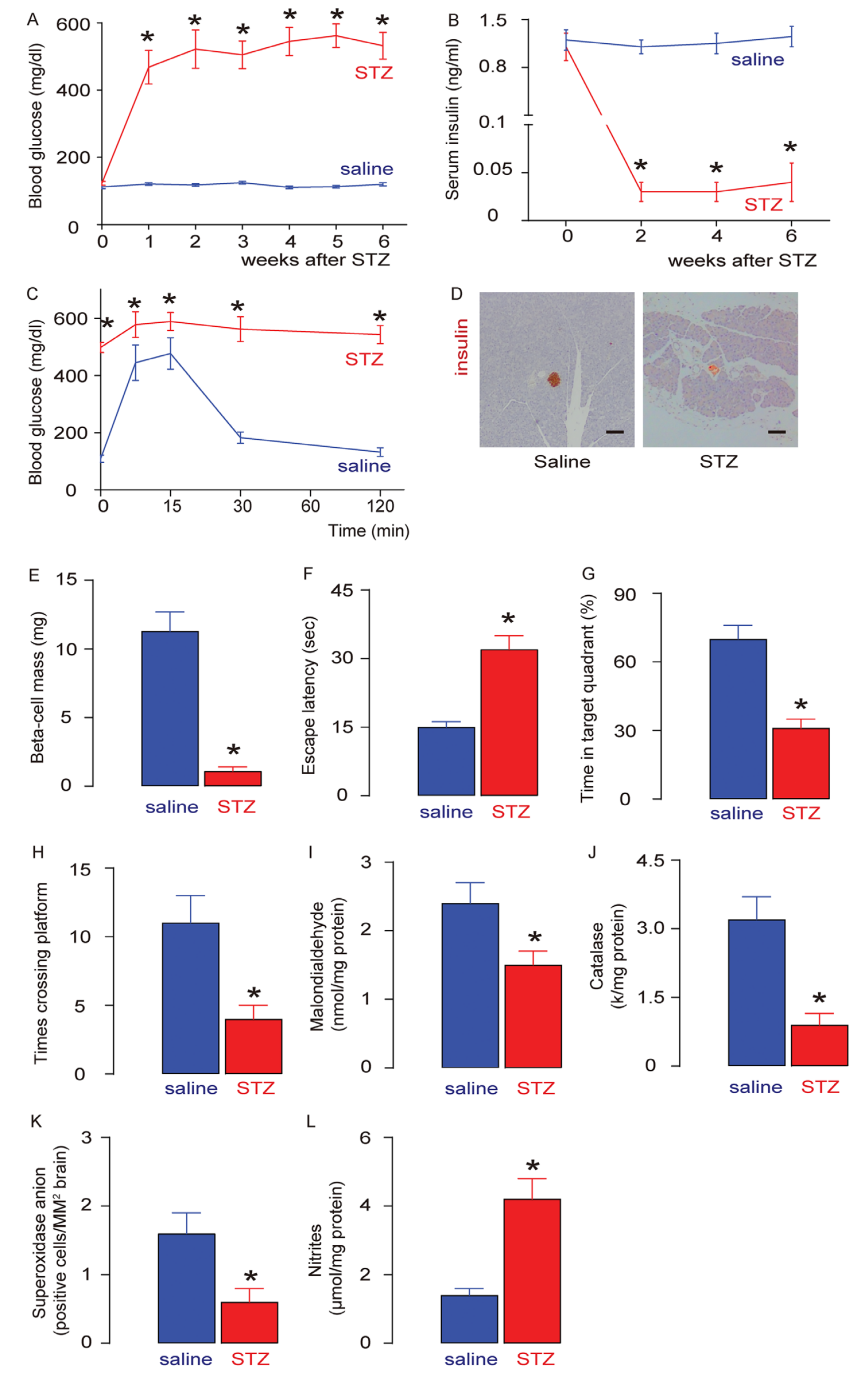
**Effects of clinical variables not shown in** **Figure 4** **on 5-year overall and disease-free survival rates.** (**A-D**) Five-year overall survival rate among the 152 rectal cancer patients, taking into account tumor differentiation, pCR status, LVI status, and the CA19-9 level. (**E-L**) Five-year disease-free survival rate among the 152 rectal cancer patients, taking into account PNI status, neoadjuvant chemotherapy cycles, T stage, pCR status, T stage, LVI status, number of neoadjuvant chemotherapy cycles, and PNI status.

The beta-cell toxin STZ was i.p. injected at a dose of 50 mg/kg body weight to induce high blood glucose levels in 18-week old Wister rats. Control rats (saline) were injected with saline. We found that STZ induced sustained fasting hyperglycemia in rats (Figure 1A), low serum insulin levels (Figure 1B), and poor glucose response (Figure 1C), likely due to loss of pancreatic beta cells (Figure 1D) that led to a significantly reduced beta-cell mass, assessed 6 weeks after STZ treatment at which point the rats were sacrificed (Figure 1E). The development of DE was assessed by an MWM assay and by analysing brain degradation markers, brain malondialdehyde, catalase, superoxidase anion-positive cells and nitrites. We found that in the MWM behavioral assay, the escape latency of STZ-treated rats was significantly prolonged, compared to saline-treated rats (Figure 1F). Moreover, the percentage of time in the target quadrant for STZ-treated rats was significantly reduced, compared to saline-treated rats (Figure 1G), and the number of times that the rats crossed the platform was significantly reduced in STZ-treated rats, compared to saline-treated rats (Figure 1H). Furthermore, we found that STZ treatment induced decreased levels of brain Malondialdehyde (Figure 1I), catalase (Figure 1J) and superoxidase anion-positive cells (Figure 1K), and induced increases in brain nitrites (Figure 1L). Together, these data suggest that STZ induces diabetes and DE in rats.

### Macrophage autophagy is decreased in STZ-treated rat brain

**Figure 2:**
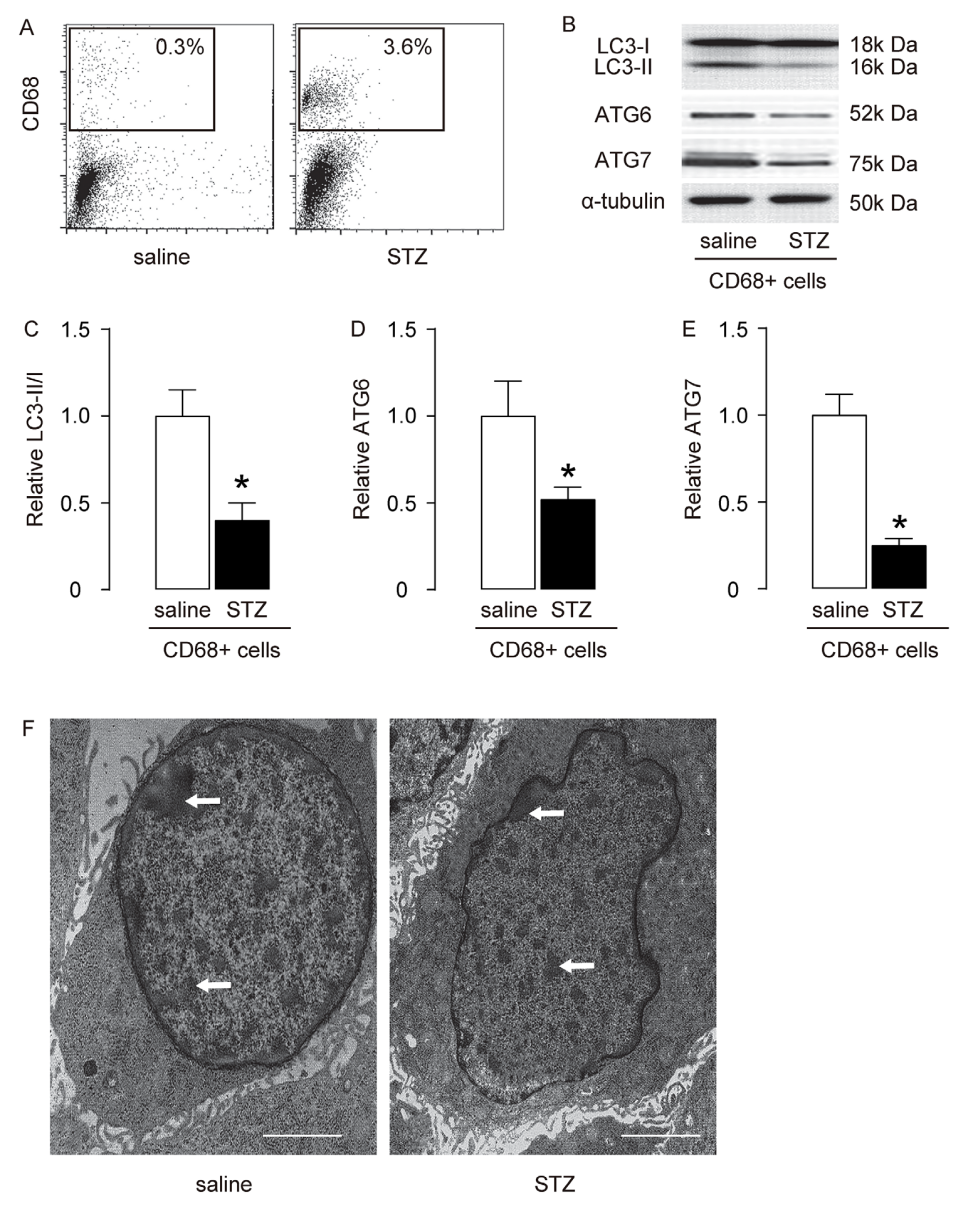
**Effects of clinical variables not shown in** **Figure 4** **on 5-year overall and disease-free survival rates.** (**A-D**) Five-year overall survival rate among the 152 rectal cancer patients, taking into account tumor differentiation, pCR status, LVI status, and the CA19-9 level. (**E-L**) Five-year disease-free survival rate among the 152 rectal cancer patients, taking into account PNI status, neoadjuvant chemotherapy cycles, T stage, pCR status, T stage, LVI status, number of neoadjuvant chemotherapy cycles, and PNI status.

Rat brains were dissociated into single cells to sort CD68+ macrophages, shown by representative flow charts (Figure 2A). The autophagy status of brain macrophages was assessed by the ratio of LC3-II to LC3-I. We found that the LC3-II/I ratio was significantly reduced in macrophages from the brain of STZ-treated rats, compared to those from saline-treated rats (Figure 2B-C). Moreover, two key autophagy-associated proteins, ATG6 and ATG7, were analyzed, with levels of both shown to be significantly decreased in macrophages from the brain of STZ-treated rats, compared to those from saline-treated rats (Figure 2B-E). The decreased autophagic activity of brain macrophages from STZ-treated rats was confirmed by EM, showing decreased number of autophagy compartments like autophagosomes (Figure 2F). Thus, macrophage autophagy is decreased in STZ-treated rat brain.

### Enhanced PI3k/AKT/mTOR signalling is detected in brain macrophages from STZ-treated rats

**Figure 3:**
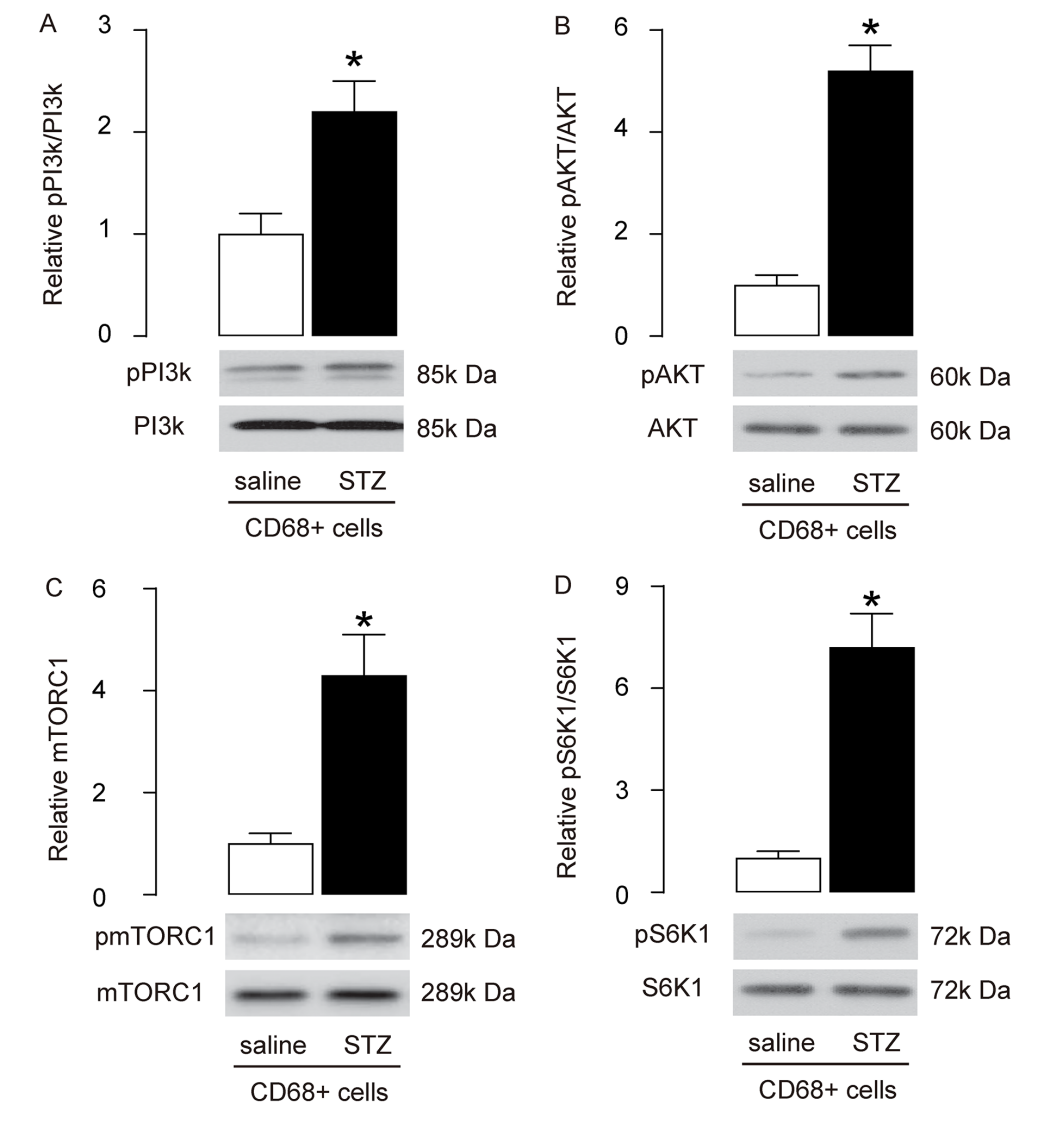
**Enhanced PI3k/AKT/mTOR signalling is detected in brain macrophages from STZ-treated rats.** (**A-D**) Western blot analysis of activation of PI3k (A), AKT (B), mTOR (C) and S6K1 (D) in brain macrophages. *p<0.05. NS: non-significant. N=10.

mTOR is a key factor that controls cell growth and autophagy. PI3k and AKT are upstream regulators of mTOR, and S6K1 is an mTOR downstream effector. Since brain macrophage autophagy is decreased in STZ-treated rats, we investigated the activities of PI3k, AKT, mTOR and S6K1 in brain macrophages. We found that the phosphorylation of both PI3K and AKT was significantly increased in brain macrophages (Figure 3A-B), which may be responsible for the activation of mTORC1 (Figure 3C), the most important component of the mTOR complex. In addition, the phosphorylation of S6k1, a key proliferation activator [24,25], was also significantly increased in brain macrophages (Figure 3D). These data suggest that STZ induces augmentation of PI3k/AKT/mTOR signalling in brain macrophages.

### STZ-induced DE is alleviated by RAP and exacerbated by CQ

**Figure 4:**
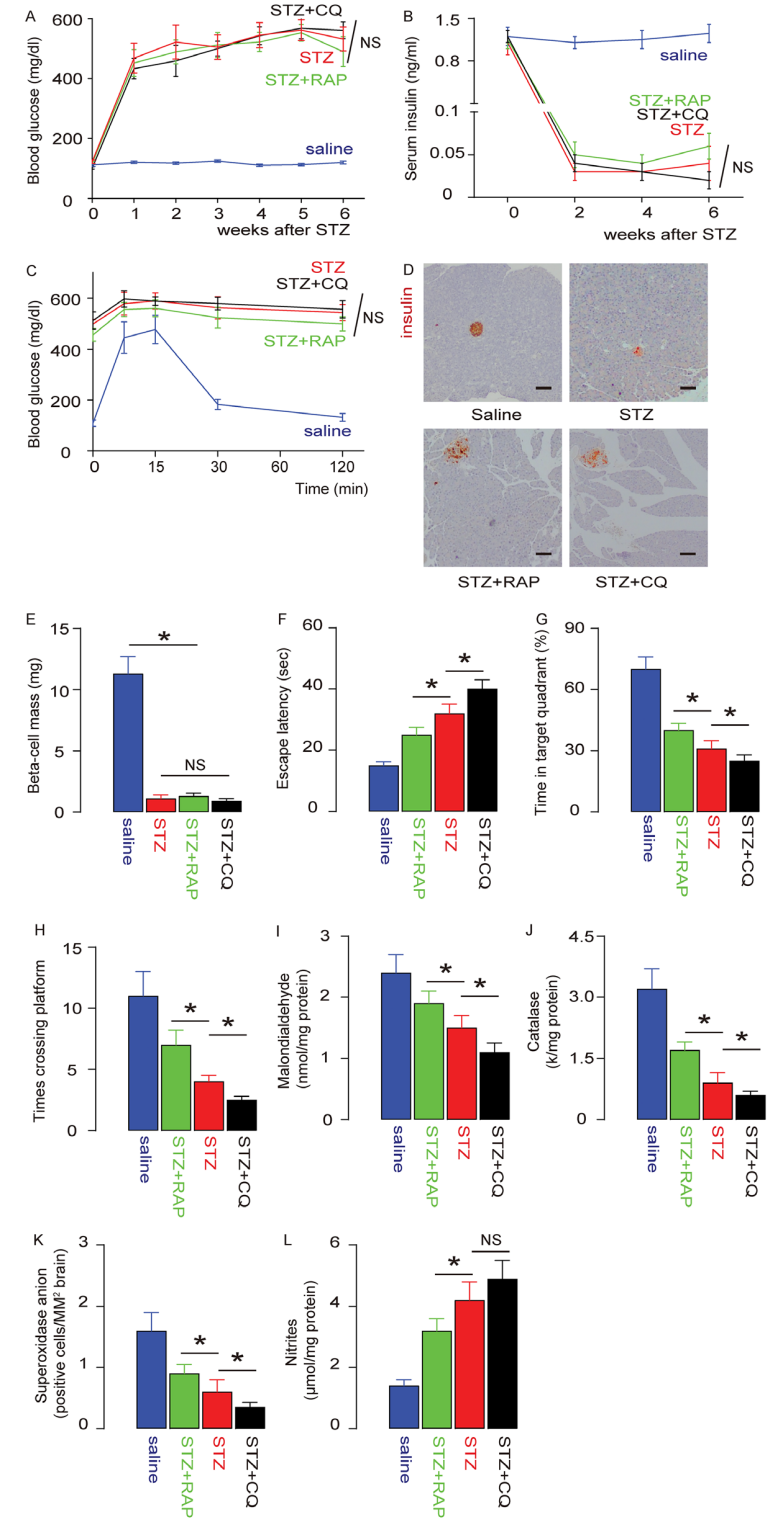
**STZ-induced DE is alleviated by RAP and exacerbated by CQ.** Rapamycin (RAP) or Chloroquine (CQ) were administered to STZ-treated rats. (**A**) Fasting glycemia in rats. (**B**) Serum insulin content. (**C**) IPGTT 6 weeks after STZ. (**D**) Representative immunohistochemistry for insulin in the tail pancreas of rats 6 weeks after STZ. (**E**) Beta-cell mass 6 weeks after STZ. (**F-H**) The development of DE was assessed by an MWM assay. (F) The escape latency. (G) The percentage of time spent in the target quadrant. (H) The number of times that the rats crossed the platform. (**I-L**) Assessment of brain degradation markers. (I) Brain Malondialdehyde. (J) Catalase. (K) Superoxidase anion-positive cells. (L) Nitrites. *p<0.05. NS: non-significant. N=10. Scale bars are 100µm.

In order to understand the role of mTOR signalling and brain macrophage autophagy in DE development, we administered either rapamycin (RAP), an mTOR inhibitor, or Chloroquine (CQ), an autophagy inhibitor, to the STZ-treated rats. We found that neither RAP nor CQ altered STZ-induced sustained fasting hyper-glycemia in rats (Figure 4A), low serum insulin content (Figure 4B), poor glucose response (Figure 4C), loss of pancreatic beta cells (Figure 4D), or significantly reduced beta-cell mass, assessed 6 weeks after STZ treatment at time of sacrificed (Figure 4E). On the other hand, in the MWM behavioral assay, the prolonged escape latency of STZ-treated rats was significantly reduced by RAP, but significantly extended by CQ, compared to saline-treated rats (Figure 4F). Moreover, the reduced percentage of time spent in the target quadrant by STZ-treated rats was significantly reversed by RAP, but significantly enhanced by CQ, compared to saline-treated rats (Figure 4G). Moreover, the reduced number of times that the rats crossed the platform in STZ-treated rats was significantly reversed by RAP, but significantly exacerbated by CQ, compared to saline-treated rats (Figure 4H). Furthermore, we found that STZ-induced decreases in brain Malondialdehyde (Figure 4I), catalase (Figure 4J) and superoxidase anion-positive cells (Figure 4K), and STZ-induced increases in brain nitrites (Figure 4L), were all attenuated by RAP, but significantly aggravated by CQ, compared to saline-treated rats. Together, these data suggest that STZ-induced DE in rats is attenuated by RAP, but significantly exacerbated by CQ.

### Macrophage autophagy is salvaged by RAP and further suppressed by CQ in STZ-treated rat brain

**Figure 5:**
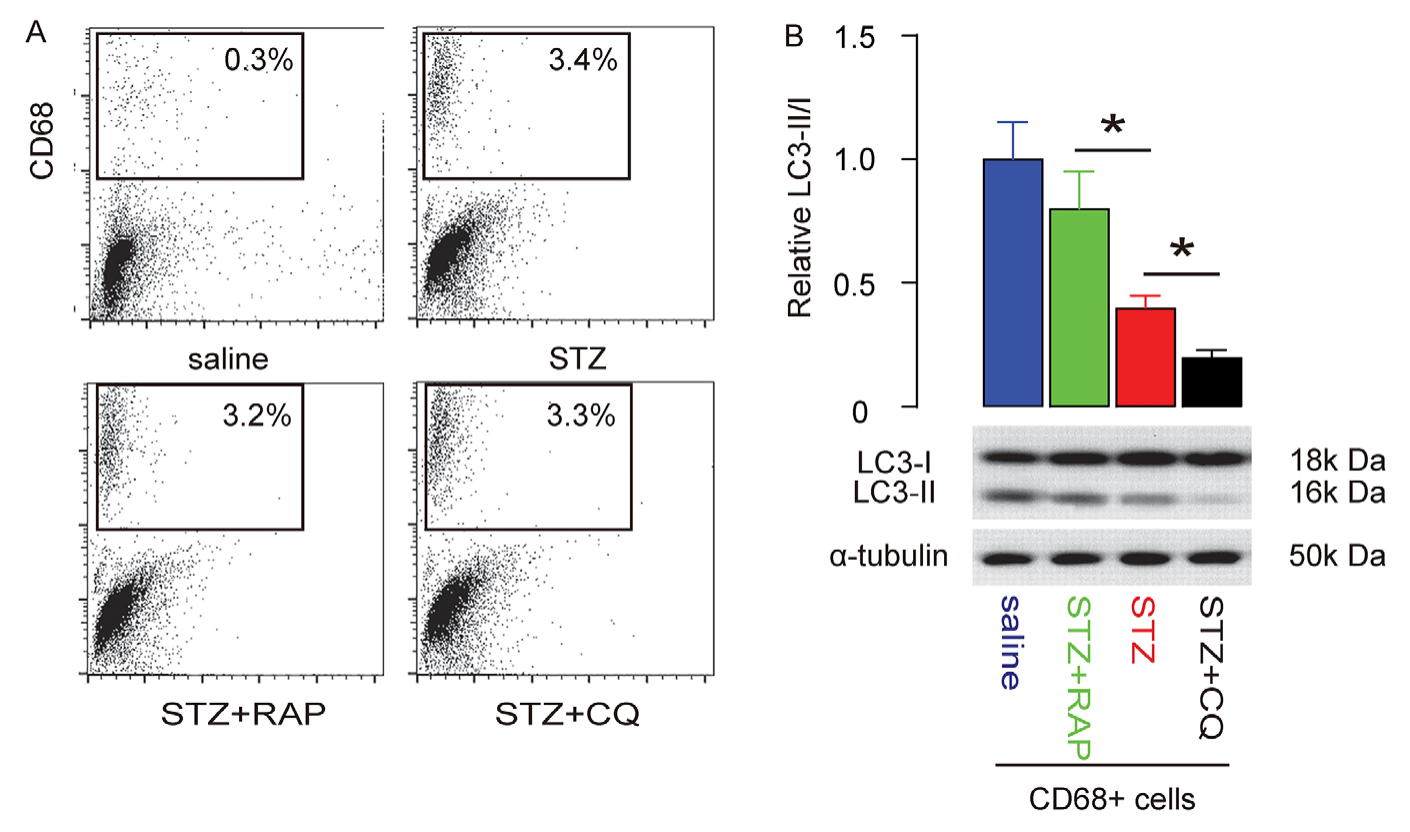
**Macrophage autophagy is salvaged by RAP and further suppressed by CQ in STZ-treated rat brain.** The rat brain was dissociated into single cells to sort CD68+ macrophages. (**A**) Representative flow charts. (**B**) Western blot for LC3 and quantification LC3-II/I ratio. *p<0.05. N=10.

Macrophages were isolated from the rat brain, shown by representative flow charts (Figure 5A). The autophagy status of brain macrophages was assessed by LC3-II/I ratio. We found that the reduced LC3-II/I ratio in brain macrophages from STZ-treated rats was significantly increased by RAP, but further reduced by CQ (Figure 5B). Thus, decreased brain macrophage autophagy is salvaged by RAP and further suppressed by CQ.

### PI3k/AKT/mTOR signalling in RAP/CQ-treated and STZ-treated rat brain macrophages

**Figure 6:**
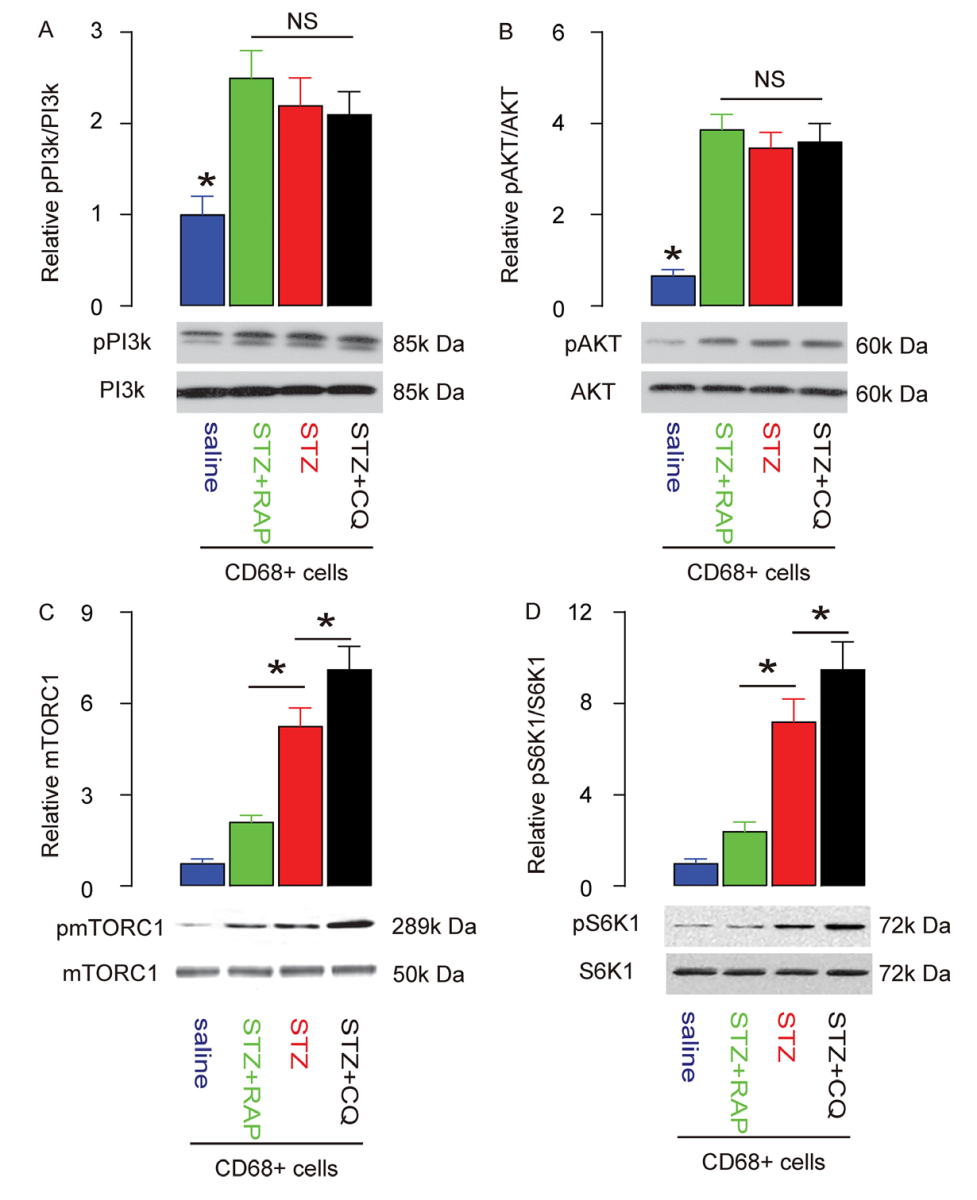
**PI3k/AKT/mTOR signalling in RAP/CQ-treated and STZ-treated rat brain macrophages.** (**A-D**) Western blot analysis of activation of PI3k (A), AKT (B), mTOR (C) and S6K1 (D) in brain macrophages from RAP or CQ treated rats. *p<0.05. NS: non-significant. N=10.

**Figure 7:**
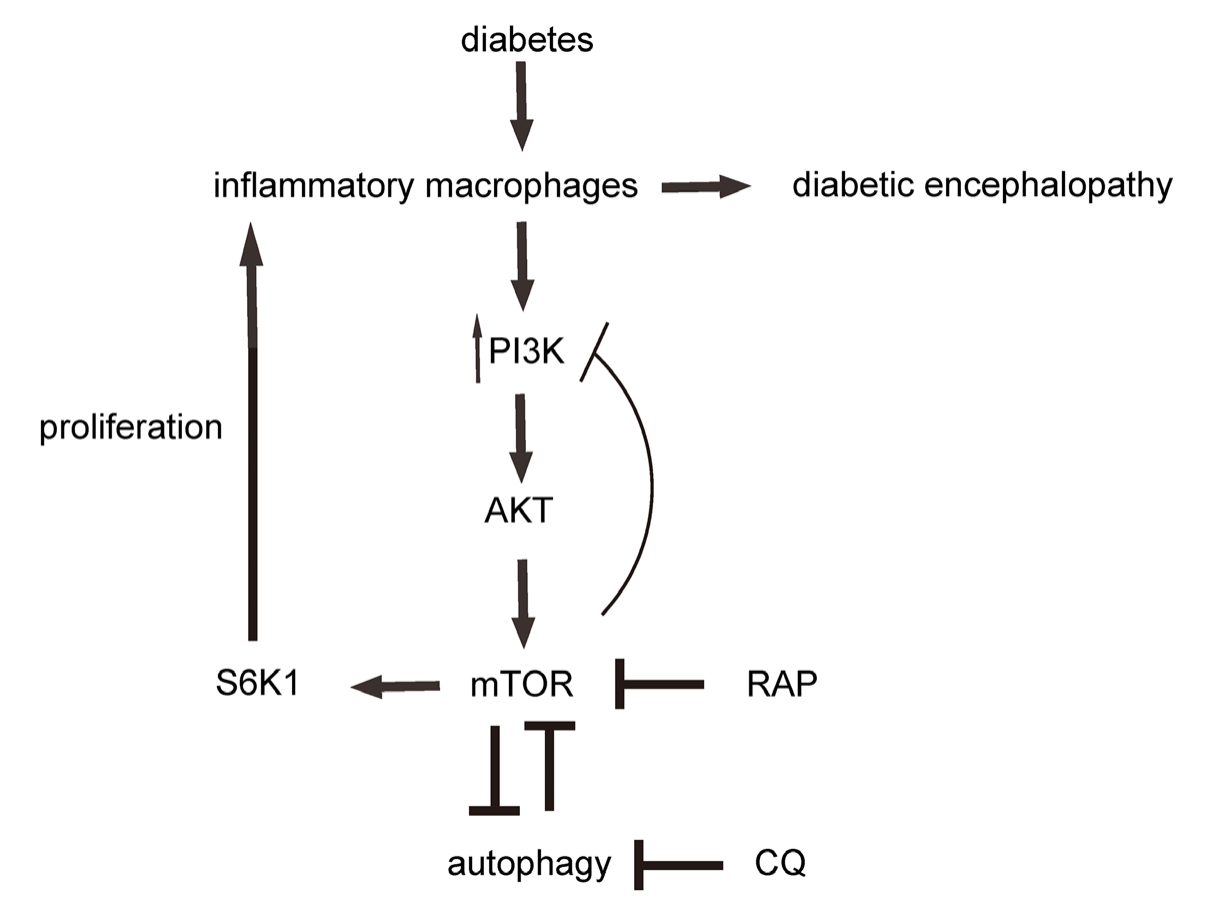
**Schematic.** Diabetes induces increases in brain inflammatory macrophages, through increased PI3k/AKT/mTOR/S6K1 signalling and suppression of autophagy. mTOR and autophagy inhibit each other. RAP inhibits mTOR. CQ inhibits autophagy.

Finally, we investigated the activity of PI3k, AKT, mTOR and S6K1 in brain macrophages. We found that the increased phosphorylation of both PI3K and AKT in brain macrophages in STZ-treated rats was not affected by either RAP or CQ (Figure 6A-B). However, the STZ-induced upregulation of mTORC1 in brain macrophages was significantly attenuated by RAP, likely through direct suppression of RAP on mTORC1 (Figure 6C). On the other hand, the STZ-induced upregulation of mTORC1 in brain macrophages was further reduced by CQ, likely through loss of the suppressive effects of autophagy on mTORC1 (Figure 6C). In addition, the phosphorylation of S6K1 was similarly influenced by RAP or CQ (Figure 6D), since S6K1 is the immediate downstream effector of mTORC1. The interaction of mTOR signalling and autophagy in brain macrophages, and their role in DE pathogenesis, are summarized in a schematic (Figure 7).

## DISCUSSION

Although chronic hyperglycemia is the main trigger for the development of DE, past studies have shown that inflammatory damage to the brain also predisposes to disease progression [8]. Recently, in a mouse STZ-induced diabetes model, we have shown the centrality of recruited and infiltrated inflammatory macrophages in the mouse brain, to the progression of DE [9]. In the current study, our first aim was to investigate if this recruitment and infiltration of inflammatory macrophages in the brain are similarly required for DE development in diabetic rats, an animal model that more closely resembles human diabetes [26]. Our second aim was to investigate the role of autophagy and mTOR signaling in brain macrophages during disease initiation and progression. Since we used CD68 to purify brain macrophages, and CD68 is expressed in both microglia and infiltrated macrophages, we did not distinguish these subtypes of macrophages in the current study. In order to differentiate the relative contribution to the development of DE, a microglia-specific marker, e.g. ionized calcium-binding adapter molecule 1 (IBA-1), may be used in the isolation of macrophages/microglia.

mTOR is a serine/threonine kinase that regulates multiple components of cellular metabolism. mTOR not only regulates cell growth and proliferation in response to a number of stimulators, but also plays an essential role in the regulation of autophagy [27]. mTOR forms two distinct signaling complexes, mTORC1 and mTORC2. mTORC1 integrates nutrient and growth factor signaling and is induced by activated RTKs-mediated downstream signaling cascades of PI3K/AKT. Once activated, mTORC1 directly activates its downstream effector S6K1 to exert its biological functions [28]. Here, we found that although it did not reach statistical significance, the phosphorylation of PI3k in brain macrophages from STZ-rats seemed to slightly increase after RAP treatment. This may result from loss of the weak inhibitory effects of mTOR on the activation of PI3K in the feedback loop, induced by RAP-induced suppression of mTOR [29]. On the other hand, although it did not reach statistical significance, the phosphorylation of PI3k in brain macrophages from STZ-rats seemed to slightly decrease by CQ. This may result from the further loss of the suppressive effects of autophagy on mTOR. The attenuation of mTORC1 by RAP appeared to result from the direct suppressive effects of RAP on mTORC1, since activation of PI3k/AKT was not apparently affected. The further increase in mTORC1 by CQ likely resulted from loss of the suppressive effects of autophagy on mTOR.

From the current study, we identified the critical role of inflammatory macrophages in DE progression. Moreover, we showed that the development of DE could be interrupted through modulation of macrophage autophagy via mTOR signaling.

## MATERIALS AND METHODS

### Protocols and animals

All rat experiments were approved by the research committee of Shanghai Jiao Tong University Affiliated Sixth People’s Hospital. Sixteen-week old female Wister rats were purchased from Charles River Labs (Beijing, China). Fasting blood glucose and serum insulin were measured as described before [9]. Streptozotocin (STZ) was intraperitoneally (i.p.) injected at a dose of 150 mg/kg body weight in sodium citrate buffer (pH=4.5) to induce sustained high blood glucose in 18-week-old female rats. Littermate rats that received injection of the same volume of saline were used as controls (saline). Serum insulin was determined with an insulin ELISA kit (R&D System, Los Angeles, CA, USA). Fasting blood glucose measurement and Intraperitoneal glucose tolerance test (IPGTT) were conducted as previously described [9]. Rapamycin (RAP) and Chloroquine (CQ) were both obtained from Sigma-Aldrich (St. Louis, MO, USA), and were i.p administrated to rats at a dose of 2mg/kg body weight, and 10mg/kg body weight, respectively, once a day for six weeks.

### Assessment of brain function

Measurement of nitrite/nitrate production, thiobarbituric reactive substances (TBARS) and catalase activity, and histological identification of superoxide anion, were performed as described before [9].

### Morris water maze (MWM)

The spatial learning and memory function of rats were assessed by the MWM assay. Briefly, a circular pool was divided into four quadrants, and a circular black platform was placed in the target quadrant, about 2.5 cm beneath the water surface. Markers in different shapes and colors were posted on the white curtain around the pool for navigation. Each rat was allowed to swim for 90 seconds to locate the hidden platform. The swimming paths of these rats were recorded with a video capture system (THDB-D5M, Terasic Technologies, Inc., Taiwan), and time spent in the target quadrant and the number of times the rats crossed the platform were calculated using recorded data.

### Flow cytometry for macrophages

Rat brains were dissociated into a single cell preparation with 0.025% Trypsin at 37°C for 35 minutes, and then incubated with PEcy5-conjugated anti-CD68 antibody (Becton-Dickinson Biosciences, San Jose, CA, USA), for fluorescence activated cell sorting (FACS). Primary rat macrophages were cultured in DMEM media (Invitrogen, CA, Carlsbad, USA) supplied with 5% fetal bovine serum (FBS, Invitrogen) and 1% penicillin/streptomycin (Invitrogen).

### Western blotting

Protein isolation and Western blotting have been described before [23]. The primary antibodies for Western Blot were anti-CD68 (1:800; Becton-Dickinson Biosciences), anti-LC3 (1:750; Cell Signaling, San Jose, CA, USA), anti-PI3K (1:750; Cell Signaling), anti-phosphorylated PI3K (pPI3K, 1:1000; Cell Signaling), anti-AKT (1:1000; Cell Signaling), anti-phosphorylated AKT (pAKT, 1:1000; Cell Signaling), anti-mTORC1 (1:1000; Cell Signaling), anti-S6K1 (1:500; Cell Signaling), anti-phosphorylated S6K1 (pS6K1, 1:1000; Cell Signaling) and anti-α-tubulin (1:1000; Cell Signaling). The secondary antibodies were HRP-conjugated anti-rabbit antibodies (Jackson ImmunoResearch Labs, West Grove, PA, USA). Western blot quantification was performed using NIH ImageJ software (Bethesda, MA, USA) using 4-5 repeats.

### Immunohistochemistry and beta cell mass

Rat pancreas and brain were dissected out and fixed in 4% paraformaldehyde for 4 hours, cryo-protected in 30% sucrose overnight, and then sectioned to 7μM. The primary antibody for insulin was guinea pig polyclonal anti-insulin (1:500; DAKO, Carpinteria, CA, USA). The secondary antibody was HRP-conjugated anti-guinea pig antibody (1:1000; Jackson ImmunoResearch Labs). The DBA method was used to develop the signals, and Hematoxylin counterstaining was performed at the end of the staining. The quantification of beta cell mass was performed as has been described before [9].

### Statistical analyses

Data were analyzed using one-away ANOVA with a Bonferroni correction, followed by Fisher’ Exact Test for comparison of two groups (GraphPad Prism, GraphPad Software, Inc. La Jolla, CA, USA), shown as the mean ± S.D. A p value < 0.05 was considered significant. NS: non-significant.
